# A Clinical Introduction to the Practice of Auscultation, and Other Modes of Physical Diagnosis; Intended to Simplify the Study of the Diseases of the Lungs and Heart

**Published:** 1846-01

**Authors:** 


ART. X.—*A Clinical Introduction to the Practice of Auscultation, and other Modes of Physical Diagnosis: intended to simplify the Study of the Diseases of the Lungs and Heart.* By H. M. HUGHES, M.D. Fellow of the Royal College of Physicians, Assistant Physician to Guy's Hospital.—*London*, 1845. 8vo, pp. 246.

THIS is a very excellent work, and cannot fail to be of great use to the attentive student. In it he will find every information requisite to direct his proceedings in the clinical study of auscultation. The author does not pretend to any originality, but every page shows that he has learnt what he teaches from the great book of nature, as well as from the writings of his predecessors. While Dr. Walshe's admirable treatise 'On the Physical Diagnosis of Diseases of the Lungs' (noticed by us vol. XV. p. 223) exists, we cannot admit, with Dr. Hughes, "that a work was wanted in which should be simply explained to the student of auscultation, not merely the origin, character, and diagnostic value of certain physical signs, but also the manner in which he should proceed to elicit them;" (Pref. p. ix;) because we believe that Dr. Walshe's treatise supplies all this in the most unexceptionable manner, and with singular clearness, conciseness, and completeness. At the same time, we are far from quarrelling with Dr. Hughes for having written and published his book. Although inferior to Dr. Walshe's in originality, in comprehensiveness, and in clearness of arrangement, and although exhibiting less of refined observation and critical sagacity, it is well calculated to fulfil the object for which it has been written. We are not sure but its very inferiority to Dr. Walshe's treatise may be a cause of its greater usefulness. There is a numerous class of students to whom the aphoristic conciseness of Dr. Walshe will be much less welcome, and even less intelligible, than the plain continuous narrative style and frequent repetitions of Dr. Hughes. And the same remark applies to the arrangement of the subjects adopted by the two authors. Dr. Walshe's plan is unquestionably much more philosophical, and is even clearer and more practical than that of Dr. Hughes; and yet this very plan,—involving as it does a greater subdivision of subjects, and thus rendering it necessary, in the working out of any one point, to refer to three or four different parts of the volume,—is likely to be a cause of puzzle and annoyance to some readers. We are therefore pleased to think that by the publication of Dr. Hughes's volume, this class of students will be conciliated, and the progress of auscultation practically extended. To the student of auscultation we would indeed say, possess yourself of both works—both will be found useful, neither superfluous. It is but justice to Dr. Hughes to add, that his work includes the subject of diseases of the heart, while Dr. Walshe's is confined to that of diseases of the lungs. Dr. Hughes's book is written with great modesty, and everywhere exhibits a becoming distrust of his own powers, and the powers of his art. He gives ample evidence of possessing that amount of knowledge which can afford to confess its deficiencies.

